# Emergent transcriptional adaption facilitates convergent succession within a synthetic community

**DOI:** 10.1038/s43705-021-00049-5

**Published:** 2021-09-01

**Authors:** Chun-Hui Gao, Hui Cao, Feng Ju, Ke-Qing Xiao, Peng Cai, Yichao Wu, Qiaoyun Huang

**Affiliations:** 1 State Key Laboratory of Agricultural Microbiology, State Environmental Protection Key Laboratory of Soil Health and Green Remediation, College of Resources and Environment, Huazhong Agricultural University, Wuhan, China; 2 Key Laboratory of Coastal Environment and Resources of Zhejiang Province, School of Engineering, Westlake University, Hangzhou, Zhejiang China; 3 School of Earth and Environment, University of Leeds, Leeds, UK

**Keywords:** Microbial ecology, Microbial ecology

## Abstract

Taxonomic convergence is common in bacterial communities but its underlying molecular mechanism remains largely unknown. We thus conducted a time-series transcriptional analysis of a convergent two-species synthetic community that grew in a closed broth-culture system. By analyzing the gene expression and monitoring the community structure, we found that gene expression mainly changed in the early stage, whereas community structure significantly changed in the late stage. The significant change of gene expression occurred even at the very beginning, which was designated as “0 h effect”, suggesting the effect of species interaction on gene expression was inevitable. Besides, the effect of interaction on gene expression has a “population effect”, which means that majority species have greater impact on gene expressions of minority species than *vice versa*. Furthermore, gene set enrichment analysis revealed that among a total of 63 unique pathways (occupying about 50% of all the metabolic pathways in both species), 40 (63%) were consistently suppressed, 16 (25%) were conditionally expressed, and only 7 (11%) were consistently activated. Overall, they were strictly regulated by both time and initial structures. Therefore, we proposed that microorganism responses and the induced gene expression changes play important roles in the process of community succession.

## Main

Convergence is a common feature of evolution and has great effect on the succession of microbial communities. For natural microbial communities such as the microbiome of gut [1], soil [2], sediment [3], rhizosphere [4], and phyllosphere [5], convergence generally means that different communities converge towards a similar species composition, which is accompanied by species loss and acquisition. Such a convergence can be reproduced in simplified synthetic communities [6–8], or even in single-species populations, in which convergence can still be achieved at sub-species level [9, 10]. Unlike the convergence of natural microbial community, those experiments carried out in a sterile laboratory environment only involves the loss of species. Specifically, the main manifestation of convergence in the synthetic community containing stably coexisting species lies in that the relative proportion of species tend to become consistent [7, 8]. Nonetheless, synthetic community opens a window for us to investigate the ecological mechanism. Previous studies of synthetic communities have revealed that the convergence of bacterial community can be regulated by pH [11], mortality [12], and particularly nutrient availability [13, 14]. Most existing studies focus on the changes in species proportions, but there is a lack of in-depth understanding of the gene expression changes driven by the community species interaction.

**Fig. 1:**
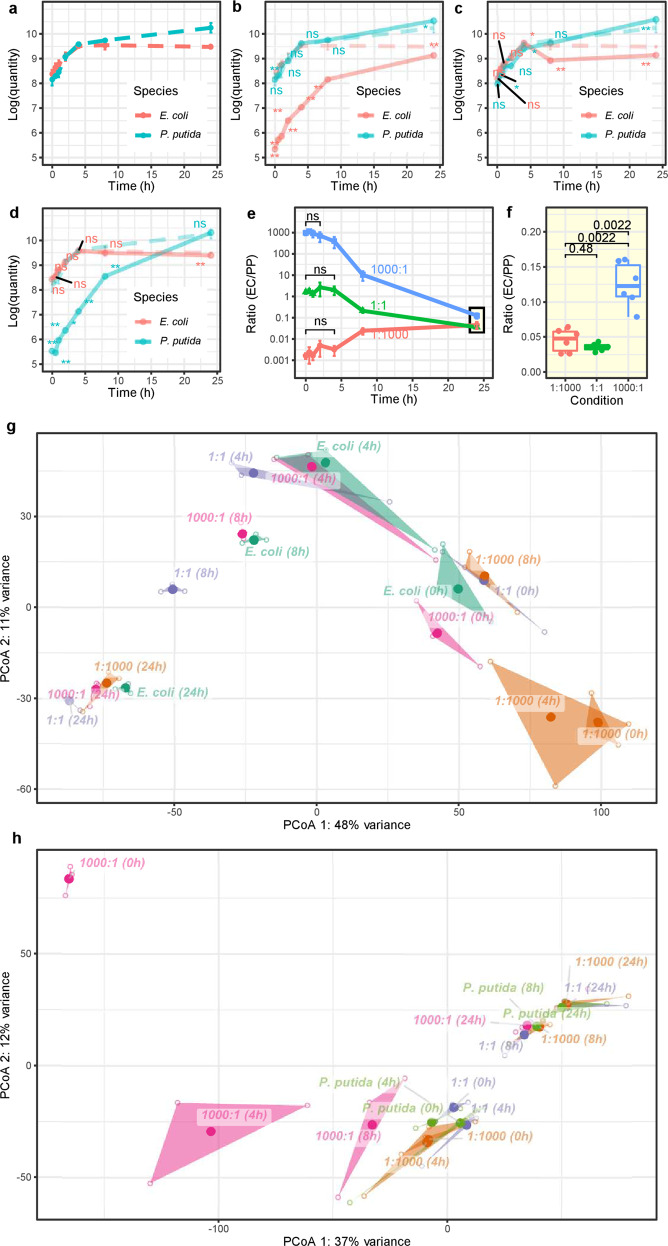
Convergence of community structure and gene expression. **a**–**d** Growth curves of *E. coli* and *P. putida* in monoculture (**a**) and the “1:1000”, “1:1”, “1000:1” cocultures (**b**–**d**). In **b**–**d** subplots, the growth curves of monocultures were placed on the background layer (dashed lines), and the significant differences in cell quantity between coculture and corresponding monoculture were shown (ns, non-significant; *p < 0.05; **p < 0.01). **e**, **f** Ratio of *E. coli* to *P. putida* in cocultures converged at 24 h. **e** Real-time ratio of EC/PP at 0, 0.5, 1, 2, 4, 8 and 24 h. **f** Analysis of the difference of EC/PP ratios (24 h) between cocultures. **g**, **h** Principal coordinate analysis (PCoA) of gene expression profiles of *E. coli* (**g**) and *P. putida* (**h**) in monoculture and three cocultures. The analysis was conducted separately for each species, with a transformed, normalized read counts using DESeq2. In the plots, the samples (3 replicates, indicated by hollow dots) with the same treatment (indicated by text annotations) form a number of triangles filled with transparency colors. The centroids of the triangles, i.e., the average values of corresponding replicates, are denoted by solid points.

In this study, we constructed a synthetic community with two model microorganisms, *Escherichia coli* K-12 (EC) and *Pseudomonas putida* KT2440 (PP), and reproduced a convergent community assembly in closed broth-culture system. In monocultures, the growth curves of both *E. coli* and *P. putida* fitted well with the bacterial growth model, and fell into a logarithmic phase at the first 4 h of bacterium culture and a stationary phase at subsequent 20 h (after the first 4 h) (Fig. 1a). When same quantities of bacteria were grown in cocultures, their quantities were basically similar to those in monocultures, particularly in the logarithmic phase (Fig. 1b–d). By contrast, the quantities of minority species in cocultures continued to increase, and they were close to the quantities in monocultures at 24 h post co-cultivation (Fig. 1b–d). Besides, statistical analysis showed that the quantities of *P. putida* in all three cocultures were overall greater than that in monoculture, while *E. coli* quantities were no more than its monoculture (Fig. 1b–d), suggesting that *P. putida* has a negative effect on the growth of *E. coli*, but *E. coli* promotes that of *P. putida*.

The ratios of two species in cocultures were initially anchored to about 10^5^:10^8^ (1:1000), 10^8^:10^8^ (1:1), and 10^8^:10^5^ (1000:1), respectively, and they converged to approximately 10^9^:10^10^ after 24 h cultivation (Fig. 1e). Specifically, the final ratios in “1:1000” and “1:1” cocultures exhibited no significant differences (Fig. 1f). Dramatical community structure changes began from 4 h, but their ratios generally displayed no significant differences between 0 h and 4 h in every coculture (Fig. 1e, except for 1000:1 at 4 h). In other words, the main manifestation of the convergence for this simple community was the coexistence of two species and the tendency to be consistent in relative proportions of species.

Convergence appeared at transcriptomic level as well. In transcriptomic analysis, we used the monoculture with the same culture time as reference to analyze the gene expression changes in cocultures. Principle coordinate analysis (PCoA) showed that the *E. coli* gene expression changes followed the similar trajectories in three cocultures over time: first from the bottom right to the upper middle, and then from the upper middle to the lower left on the coordinate system composed of the first two eigenvectors (Fig. 1g). The *E. coli* gene expression dispersion was the highest at 0 and 4 h in the “1:1000” coculture (Fig. S1a). Likewise, the gene expression trajectory of *P. putida* was from the top left to the bottom middle, and then to the top right over time (Fig. 1h), and the *P. putida* gene expression dispersion was the highest at 0, 4, and 8 h in the 1000:1 coculture (Fig. S1b). Overall, the differences in gene expression under different cultures was larger in the early stage, but gradually disappeared in the late stage. As a result, convergence of gene expression was finally observed for both *E. coli* and *P. putida* at 24 h. Correspondingly, differentially expressed genes (DEGs) were mainly identified in early stage (Fig. S2, in the first 8 h).

Generally, the majority species had more significant effect on the gene expression of minority species in cocultures. For example, the minority *E. coli* had more DEGs than *P. putida* in the “1:1000” coculture (Fig. S3), and the minority *P. putida* had more DEGs than *E. coli* in the “1000:1” coculture (Fig. S3). Overall, the gene expression of *E. coli* was more affected by *P. putida*, and the total number of all DEGs in *E. coli* was approximately four folds as much as that in *P. putida* (Fig. S2).

Furthermore, gene expressions in cocultures were most affected during the transition stage. In the “1:1000” coculture, 139 genes were up-regulated, and 274 genes were down-regulated at 8 h in *E. coli* (Fig. S2a). In the “1000:1” coculture, 12 genes were up-regulated, and 73 genes were down-regulated at 8 h in *P. putida* (Fig. S2b). Besides, the number of DEGs varied greatly with cultivation time (Fig. S3). For instance, a total of 57, 69, 306, and 19 DEGs were found at 0 h, 4 h, 8 h and 24 h in *E. coli* in the “1:1000” coculture, respectively (Fig. S3). Although some of DEGs were shared by samples collected at different time points, most of them were specific to cultivation time (Fig. S3). These data implied that the bacterial gene expressions were gradually optimized in the progress of community convergence.

**Fig. 2:**
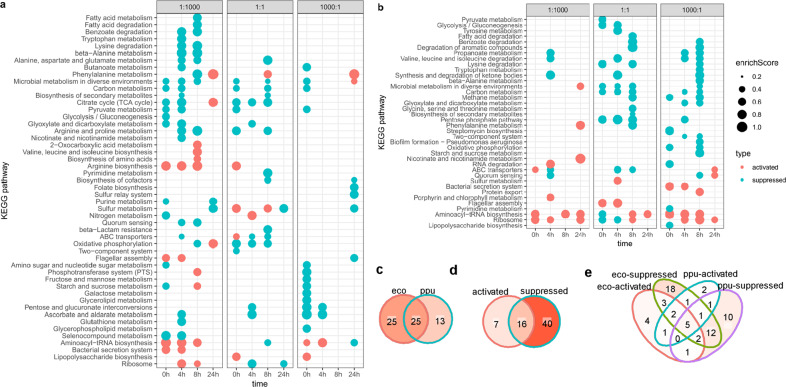
Functional enrichment in community convergence. **a**, **b** GSEA results of cocultures in *E. coli* (**a**) and *P. putida* (**b**). The *x*-axis represents sampling time of 0 h, 4 h, 8 h and 24 h, and the *y*-axis represents KEGG pathways. Pathways were sorted by Jaccard similarity of pathways containing genes. The dot size represents the absolute value of enrichment score (the larger the value, the greater the change of gene expression of the corresponding pathway), and the dot color represents whether the pathway is activated (red) or suppressed (cyan). **c**–**e** Overlap of GSEA pathways. **c** Overlap of *E. coli* (eco) and *P. putida* (ppu) pathways. d Overlap of activated and suppressed pathways. **e** Overlap of *E. coli-*activated/suppressed and *P. putida-*activated/suppressed pathways.

GSEA was employed to reveal small but evidenced expression changes, and the results showed that a total of 50 and 38 pathways were significantly changed in *E. coli* and *P. putida*, respectively (Fig. 2). These pathways accounted for 61% (50/81, in *E. coli*) and 46% (38/82, in *P. putida*) of all the investigated metabolic pathways, respectively. Overall, about half of the metabolic pathways in both species are involved in the regulation of community convergence.

The 50 pathways found in *E. coli* fell into five groups (Fig. 2a). The 18 pathways in the first group (from “fatty acid metabolism” to “nicotinate and nicotinamide metabolism”, top down, the same below) were consistently suppressed from 0 h to 8 h (except phenylalanine metabolism in “1:1” coculture), but several were activated at 24 h. By contrast, the 4 pathways in the second group (from “2-oxocarboxylic acid metabolism” to “arginine biosynthesis”) were all activated in cocultures. The 17 pathways in the third group (from “pyrimidine metabolism” to “starch and sucrose metabolism”) were opportunistically expressed across cocultures. Besides, the 7 pathways in the fourth group (from “galactose metabolism” to “selenocompound metabolism”) were constantly suppressed in all the cocultures. However, the suppressions of these 7 pathways were generally found in the “1000:1” coculture, which was different from the 18 pathways in the first group (suppressed in the “1:1000” coculture). The 4 pathways in the fifth group (from “aminoacyl-tRNA biosynthesis” to “ribosome”) were activated in the “1:1000” coculture, but conditionally expressed (activated or suppressed) in the other cocultures.

The 38 enriched pathways in *P. putida* fell into two groups (Fig. 2b). The first group contained 29 pathways (from “pyruvate metabolism” to “quorum sensing”). They were generally suppressed in the “1:1” and “1000:1” cocultures, but were mainly not changed in the “1:1000” coculture, except five pathways were activated at 24 h and two pathways were activated in 0 h and 4 h, respectively. The second group contained 9 pathways (from “sulfur metabolism” to “lipopolysaccharide biosynthesis”), and they were mostly activated in cocultures, except that two of them were suppressed at 0 h and 4 h in the “1:1” and “1000:1” cocultures.

Taken together, among all the 63 unique pathways, 25 are both identified in *E. coli* and *P. putida*, while 25 and 13 pathways are specific to them, respectively (Fig. 2c). Furthermore, 40 are only suppressed in cocultures, occupying the majority, 7 of them are only activated, and 16 of them are conditionally expressed (Fig. 2d). Overall, these data suggest that the gene expressions in cocultures are mostly suppressed, compared with those in monocultures. However, the suppressed pathways vary from species. For example, 18 and 10 pathways are specific to *E. coli* and *P. putida*, respectively (Fig. 2e). Besides, 4 and 2 pathways are found to be specifically activated in *E. coli* and *P. putida*, respectively. These data further indicate that the changing patterns for the two species are unique. Nonetheless, 12 suppressed pathways and one activated pathway are shared in *E. coli* and *P. putida* (Fig. 2e). Specifically, five pathways, including aminoacyl-tRNA biosynthesis, ABC transporters, ribosome, phenylalanine metabolism, and microbial metabolism in diverse environments, are conditionally suppressed/activated in both *E. coli* and *P. putida* (Fig. 2e).

Two-species coculture is a classic and ideal system to study population dynamics and inter-species interactions. For instance, by analyzing the population dynamics of two *Paramecium*, the competitive exclusion principle has been summarized [15]. Although this study only used two model microorganisms, our conclusions can theoretically be extended to typical microbial communities under similar conditions. However, it should be noted that this research was carried out under such a restricted condition: firstly, these two species can coexist stably in the cultivation; secondly, the community composition of these two species tends to become similar after the co-cultivation. On this basis, we can draw the following three conclusions by analyzing the patterns of gene expression.

First is what we defined “the 0 h effect”, which means that gene expression started to change at the very beginning of cultivation in community (Fig. 1 and Figs. S2, S3). Since both *E. coli* and *P. putida* are highly adaptive bacteria species that can survive in multiple environments, both of them are assumed to obtain enough nutrients while grown together in the rich medium. Therefore, it has been hypothesized that the two species might have little influences on each other’s gene expression in cocultures, especially in the early stage when the total population is relatively small. However, our results overturned this hypothesis. Instead, the gene expression changes are even more pronounced in the early stage when nutrients are the most abundant. In addition, we have also found that even when the quantities of cells in the co-cultures are comparable to those of monocultures, the interaction between species still had a significant impact on gene expression (Figs. 1 and 2). The results suggest that bacterial interaction is inevitable in cocultures, regardless of nutrient supply (niche condition) or the density of cells.

Second is the “population effect”, which means that gene expressions of minority species are more significantly influenced by majority species in cocultures. In the *E. coli* and *P. putida* cocultures, the proportion of *P. putida* in the final community is very large, accounting for approximately 90% or above (Fig. 1). At the same time, the expression of *P. putida* genes in co-culture was less affected by *E. coli*. All these results indicate that *P. putida* exhibits the growth advantage over *E. coli*. However, *P. putida* may still be the minority of a community in the “1000:1” coculture, thus *P. putida* is more effected by *E. coli* at early stage (Fig. 2).

Third is that gene expressions are strictly regulated by both time and initial structures. Compared with those growing in monoculture, the organisms growing in cocultures suppress more pathways than activate, and up to 16 pathways are suppressed throughout convergent process in both *E. coli* and *P. putida* (Fig. 2). These suppressed pathways are related to carbon metabolism, fatty acids metabolism, biosynthesis of secondary metabolites, and so on. By contrast, the activated pathways are limited and are related to biosynthesis of amino acids, RNA degradation, bacterial secretion system, and so on. It seems that cell metabolism is channeled to gene translation and secretion by these changes.

Taken together, we analyzed the gene expression changes throughout the convergent process and identified three general patterns in transcriptional regulation. Since changes in community structure are later than changes in gene expressions and our experimental settings are based on a closed broth-culture system, it could be concluded that convergence of the community is induced by changes in gene expression. Therefore, these patterns provide an insight into the molecular basis of the convergence of bacterial communities.

## Methods

### Experimental workflow

A two-species coculture system was established by previously described procedures using two bacteria at the initial ratio of *E. coli* to *P. putida* (EC/PP) of 1:1000, 1:1, 1000:1, respectively [8]. All strains were routinely grown in LB at 28 °C in shaking bed (180 rpm). Samples were collected in at least triplicates at 0, 0.5, 1, 2, 4, 8, and 24 h post cultivation. Afterwards, bacterial growth was monitored using species-specific quantitative PCR, and gene expressions were analyzed using high-throughput mRNA Sequencing (RNA-seq).

Standard strand-specific RNA-seq library construction protocol was followed and sequencing was carried out on the HiSeq X-Ten platform. After quality control, 2.6–3.9 M paired reads, and 3.9–5.9 G base pairs were obtained from each sample. High-quality reads were aligned against the *P. putida* and *E.coli* reference genome using hisat2 [16]. All the reads were used to align the two genome sequences together, and the overall alignment rates ranged from 97.23% to 98.43%. Afterwards, reads counts of every gene were obtained with htseq-count algorithm with default settings [17]. The reads count table was subsequently divided into two parts, one for *E. coli* and the other one for *P. putida*, and these two parts were used as DESeq2 input, separately [18]. When we identified differentially expressed genes (DEGs) in cocultures, the corresponding monoculture with the same sampling time was used as reference, and the DEGs with a log2-transformed fold change > 1 (or < −1) and adjusted p-value < 0.05 were identified.

### Functional enrichment analysis

Functional enrichment analysis was performed using ClusterProfiler with the KEGG pathway annotations [19]. Gene Set Enrichment Analysis (GSEA) [20] was conducted to detect small but evidenced expression changes. Dotplot was employed to visualize the result of functional enrichment analysis. In order to sort the pathway in axis, the Jaccard similarities of pathways were calculated, and then used to construct a neighbor joining tree (see data availability). Venn plots were generated by the R package ggVennDiagram (https://CRAN.R-project.org/package = ggVennDiagram).

## Supplementary information

Supplementary information

## Data Availability

R=aw data of qPCR experiments, together with the analytical codes in R, have been deposited to GitHub repository http://github.com/gaospecial/community-convergence. Reads of RNA-seq have been deposited into Sequence Read Archive (SRA) database under the accession of PRJNA675662.
