# Supplementary material for: Emergent transcriptional adaption facilitates convergent succession within a synthetic community
Source: ISME Commun. 2021 Sep 1;1:46. doi: 10.1038/s43705-021-00049-5 (PMC9723742; doi:10.1038/s43705-021-00049-5)
Supplement: Supplementary file 1 — Supplementary information [file 43705_2021_49_MOESM1_ESM.pdf]

Figure S1

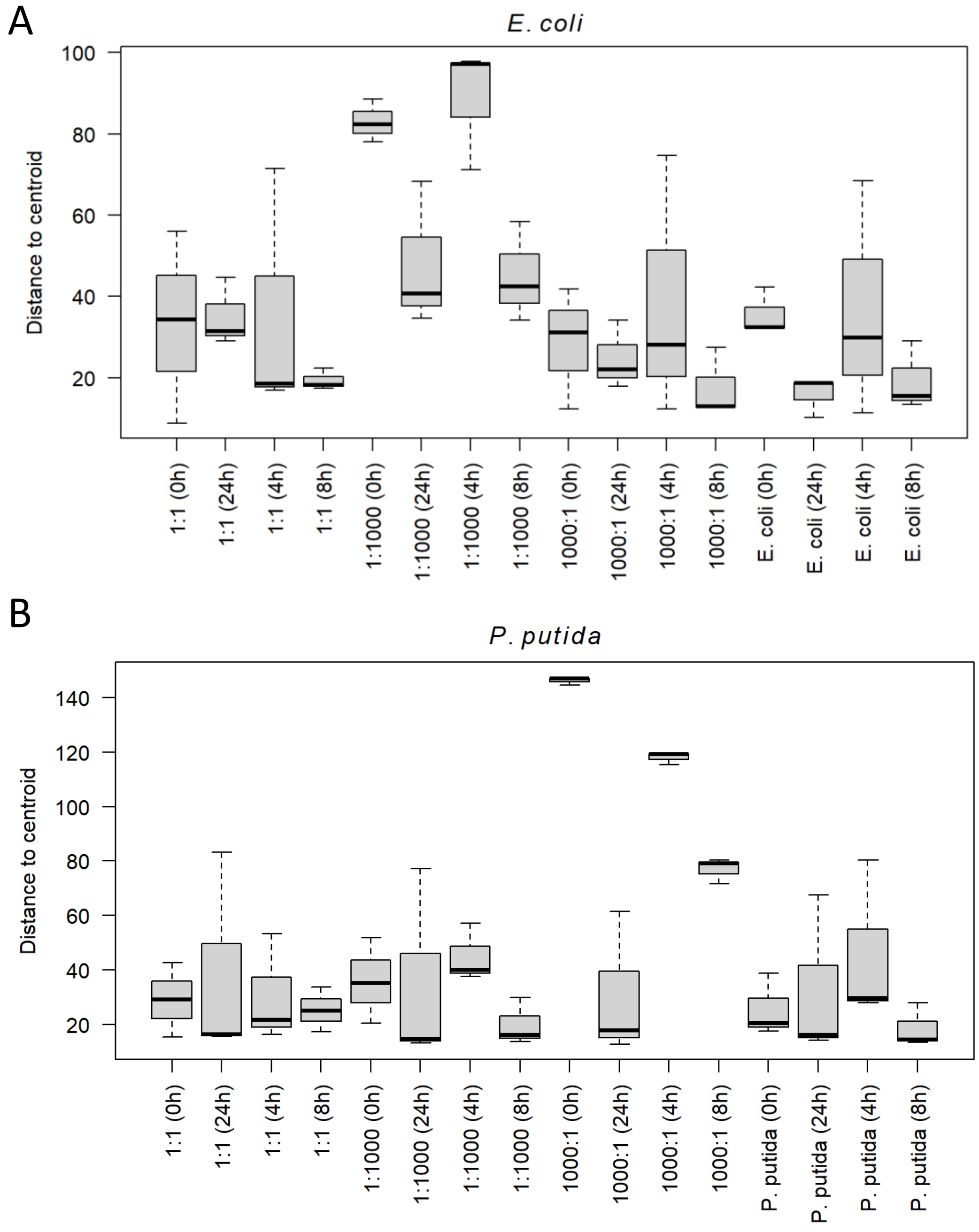

Figure S1. Comparison of beta-dispersion of gene expression in *E. coli* (A) and *P. putida* (B) in different samples. This plot is related to Figure 1g-h. X-axis represents the treatment of sample, and y-axis is the distance to centroid of replicates.

Figure S2

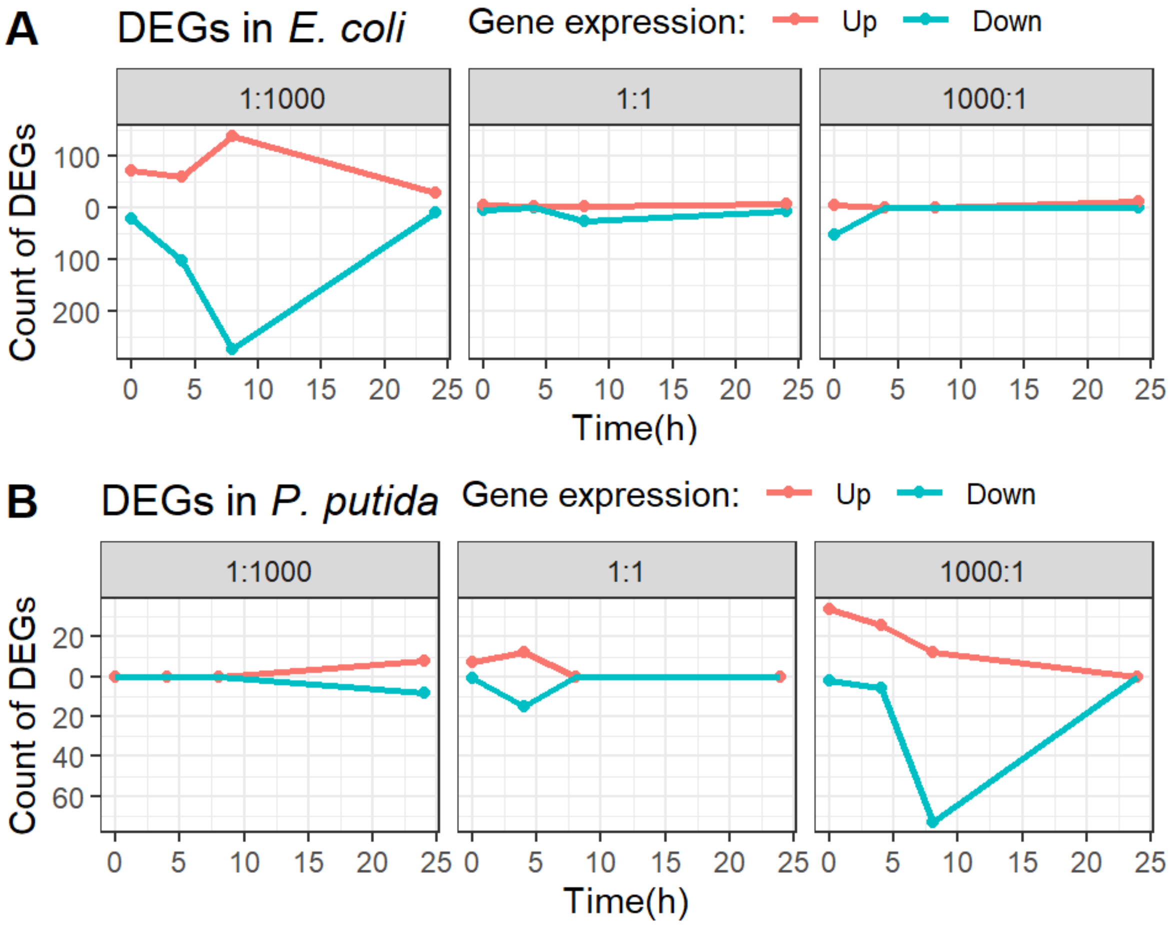

Figure S2. Counts of differentially expressed genes (DEGs) in three cocultures, in *E. coli* (A) and *P. putida* (B). The DEGs were identified by comparison with the corresponding monoculture at the same time. Up- and down-regulation of genes were colored by red and cyan, respectively.

Figure S3

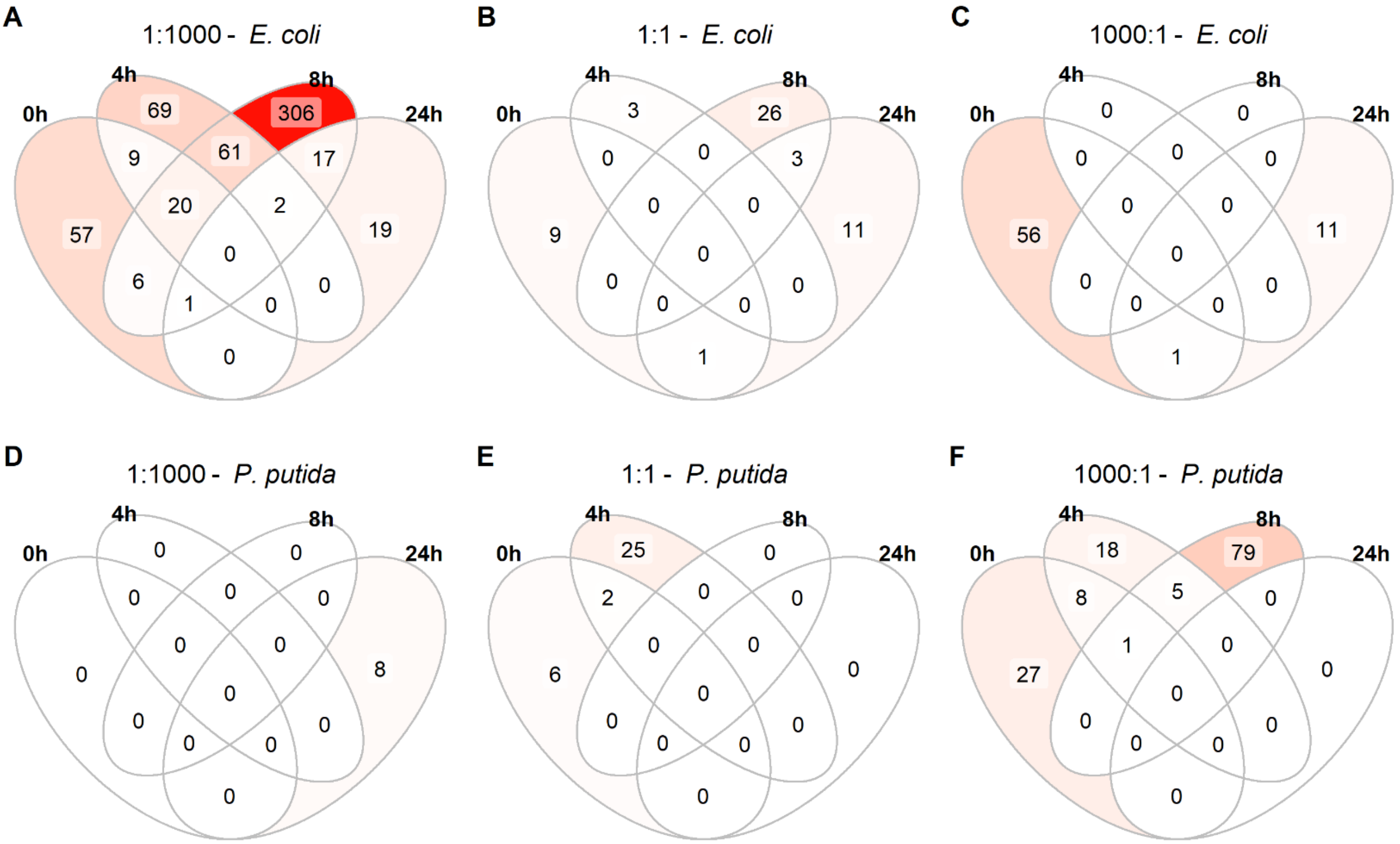

Figure S3. Venn plots showing the overlap of DEGs in three cocultures in *E. coli* (A-C) and *P. putida* (D-F).
